# The Tactile-Visual Conflict Processing and Its Modulation by Tactile-Induced Emotional States: An Event-Related Potential Study

**DOI:** 10.3389/fpsyg.2021.616224

**Published:** 2021-04-14

**Authors:** Chengyao Guo, Nicolas Dupuis-Roy, Jun Jiang, Miaomiao Xu, Xiao Xiao

**Affiliations:** ^1^School of Public Health and Management, Chongqing Medical University, Chongqing, China; ^2^Research Center for Medicine and Social Development, Chongqing Medical University, Chongqing, China; ^3^Innovation Center for Social Risk Governance in Health, Chongqing Medical University, Chongqing, China; ^4^Département de Psychologie, Université de Montréal, Montréal, QC, Canada; ^5^Department of Basic Psychology, School of Psychology, Third Military Medical University, Chongqing, China

**Keywords:** tactile-visual pairing task, event-related brain potentials, emotional state, ND420-620, the cross-modal conflict processing

## Abstract

This experiment used event-related potentials (ERPs) to study the tactile-visual information conflict processing in a tactile-visual pairing task and its modulation by tactile-induced emotional states. Eighteen participants were asked to indicate whether the tactile sensation on their body matched or did not match the expected tactile sensation associated with the object depicted in an image. The type of tactile-visual stimuli (matched vs. mismatched) and the valence of tactile-induced emotional states (positive vs. negative) were manipulated following a 2 × 2 factorial design. Electrophysiological analyses revealed a mismatched minus matched negative difference component between 420 and 620 ms after stimulus onset in the negative tactile-induced emotional state condition. This ND420-620 component was considered as a sign of the cross-modal conflict processing during the processing of incongruent tactile-visual information. In contrast, no significant mismatched minus matched negative difference component was found in the positive tactile-induced emotional state condition. Together, these results support the hypothesis that a positive emotional state induced by a positive tactile stimulation improves tactile-visual conflict processing abilities.

## Introduction

Cross-modal information processing occurs when perception involves interactions between two or more different sensory modalities (Gómez et al., 2004). For example, people sometimes rely on visual appearance and the voice of a person to know if she or he is an acquaintance. In other situations, people may rely on auditory and tactile signals, such as when they want to shut off an alarm clock in a dark room. Sniffer dogs used by rescue teams make use of their acute sense of smell and audition to find survivors in the rubble. Cross-modal information processing thus plays a central role in the production of adapted behaviors.

Cross-modal information processing includes both cross-modal information integration and cross-modal conflict processing (Saito et al., 2003; Nakashita et al., 2008). Cross-modal conflict processing specifically concerns processes that efficiently inhibit a signal in order to resolve an interference between modalities (Wang et al., 2011). A pairing task is a paradigm commonly used to study the brain mechanism underlying cross-modal conflict processing. In a classic pairing task, subjects are asked to compare stimuli from different sensory channels and indicate whether they match or not (Yin et al., 2008; Wang et al., 2011; Xiao et al., 2011a). For example, in the study of Xiao et al. (2011a), subjects had to indicate whether the taste of a gustatory stimulus matched or did not match the taste of the food item depicted in an image.

It is well-known that event-related potentials (ERPs) is an efficient technique to measure perceptual and cognitive processes prior to a behavioral response. In this technique, the brain electrical activity is recorded during the presentation of external stimuli, which allows for the precise characterization of the time course of neural activation during cross-modal conflict processing. For the last few years, researchers have recorded ERPs during pairing tasks to investigate the neural bases of cross-modal conflict processing (Yin et al., 2008; Wang et al., 2011; Xiao et al., 2011a). Electrophysiological studies on this subject have been conducted in several perceptual settings, and most have reported significant mismatched–vs.–matched negative difference waveforms (ND) usually occurring between 400 and 600 ms after stimulus onset. This negative component is believed to be associated with the conflict processing of cross-modal information. For example, Yin et al. (2008) used EEG to investigate the neural correlates of the audiovisual cross-modal conflict processing recorded in an audiovisual pairing task. Participants had to indicate if the written Chinese character presented at the center of the screen matched or did not match the uttered Chinese character. Yin et al. (2008) found a mismatched minus matched negative difference component at 490 ms (ND490) and they hypothesized that this component could be involved in the resolution of the conflict between visual and auditory information. Wang et al. (2011) used a pairing task to investigate the brain mechanism of audiovisual conflict processing in children. Participants were shown a written target word while a distractor word was uttered through loudspeakers. Immediately after, a written probe word—which could be congruent or incongruent with the target word—was displayed on the screen, and participants were asked to indicate if the probe and the target word matched or not. ERP data revealed an audiovisual mismatched minus matched negative component (ND550). In 2011, Xiao et al. investigated the ERPs elicited in a taste-visual pairing task. Participants were instructed to indicate whether the taste (sweet or sour) associated with the food item depicted in an image matched the taste (sweet or sour) of the substance on their tongue. Xiao et al. (2011a) found that mismatched answers elicited a more negative ERP deflection between 400 and 600 ms than matched answers. They postulated that this component was involved in the processing of incongruent cross-modal information. Although previous studies have shed some lights on the neural mechanisms of cross-modal conflict processing, none has specifically examined, to the best of our knowledge, the tactile-visual cross-modal conflict processing with ERPs.

Transitory affective states can have an impact on our behavior by interacting with our cognitive processes (Mitchell and Phillips, 2007). For example, a car driver receiving bad news while driving might not be able to process cross-modal information as efficiently as he/she normally does, and this could cause accidents. It is well-known that sensory stimulations can induce a positive (or negative) emotional state (Macht and Mueller, 2007; Chapman et al., 2009), however, the impact of these transitory affective states on cross-modal conflict processing has not yet been investigated extensively. In the Xiao et al. (2011a) electrophysiological study mentioned above, the positive and negative sensory stimuli were intermixed within the same condition. This may have canceled the impact of sensory-induced emotional states on cross-modal conflict processing. To address this issue, Xiao et al. (2018) directly manipulated the valence of the gustatory context (i.e., appetitive or aversive) during a taste-visual cross-modal pairing task and used fMRI to further understand the role of sensory-induced emotional state on the conflict processing of cross-modal information. A statistical contrast between the mismatched and the matched conditions revealed a significant activation of the middle frontal gyrus in the negative but not in the positive gustatory condition. This suggested that the neural response involved in cross-modal conflict processing could be modulated by sensory-induced emotional states. Based on these results, the authors proposed that the positive emotional state induced by the appetitive gustatory stimulation could have increased cognitive flexibility and improved cross-modal conflict processing abilities. Previous studies have confirmed that tactile stimulations could induce a positive (or negative) emotional state. For instance, rubbing soft and smooth materials on one's skin will usually produce pleasant feelings while rubbing it on sharp or coarse materials will usually produce the opposite effect (Major, 1895; Essick et al., 2010). This is also consistent with criteria used to select bed sheets: people usually choose soft and smooth materials to induce a pleasant and relaxed emotional state, which could in turn help them fall asleep. To the best of our knowledge, no evidence of a tactile-induced emotional state effect has been shown in the literature on tactile-visual cross-modal conflict processing. The goals of the current experiment were 2-fold: (1) to investigate the event-related brain potentials associated with the tactile-visual cross-modal conflict processing; (2) and to examine the effect of positive/negative tactile-induced emotional states on tactile-visual cross-modal conflict processing using high-density (64 channels) ERP recording.

Past studies have shown that the negative difference (ND) waveform (mismatched minus matched) between 400 and 600 ms post-stimulus was related to the conflict processing of cross-modal information. For this reason, we expected a similar ND waveform (mismatched minus matched) in the current study. Given previous studies showing that a positive sensory-induced emotional state can improve cross-modal conflict processing abilities, we expected the valence of the tactile-induced emotional states to interact with cross-modal conflict processing abilities. Following Xiao et al. (2018) findings, we expected a facilitatory effect of the positive tactile-induced emotional state condition on cognitive control abilities that would translate into a reduction of the electrophysiological activity related to cross-modal conflict processing. At the ERP level, we thus expected a lower amplitude of the mismatched minus matched ND waveform in the positive than in the negative tactile-induced emotional state condition.

## Materials and Methods

### Participants

Seventy university students were recruited in the Chongqing area (China). At the beginning, participants needed to rate their emotional state on a seven-point Likert scale ranging from 1 (highly negative) to 7 (highly positive), after being exposed to each of the six visual stimuli, which included six images of objects: needle, cactus, bayonet, thick quilt, pillow and cushion. Sixty-eight of them rated all the images as neutral. These 68 participants then had to rate their emotional state after being exposed to the two tactile stimuli. In the “negative” condition, a cactus cladode was placed on the palm of the participant's left hand, and a half-kilo wood brick was positioned on top of the cactus to exert a constant pressure. In the “positive” condition, a pillow with a soft cotton cover was placed between the participant's back and the back of the chair on which he/she was sitting. Forty-nine participants rated the pillow procedure as being highly positive and the cactus procedure as being highly negative. Twenty of the forty-nine participants told us that they could endure the discomfort caused by the cactus procedure for the whole duration of the experiment. Two participants had to quit the study due to scheduling conflicts. Finally, the remaining 18 participants (9 male and 9 female) could finish the rest of the study. All participants were healthy, right-handed, and had normal or corrected to normal vision. None of the participants reported any skin allergy.

Note that the procedures used to produce the two tactile sensations were calibrated based on a small pilot study with 10 participants who did not participate in the final study. These participants had to rate, on seven-point Likert scales, (1) their level of familiarity with each stimulus, (2) their emotional state after being exposed to each stimulus, and (3) their perceived intensity of each stimulus. Results indicated that there were no noticeable differences between the two chosen tactile stimuli in terms of familiarity and perceived intensity. The mean perceived intensity of the cactus experience was 5.2 ± 0.63 and the mean intensity of the pillow experience was 4.9 ± 0.88. The mean level of familiarity on the cactus experience was 5.1 ± 0.74 and the mean level of familiarity on the pillow experience was 5.6 ± 0.52. All participants rated the pillow experience as highly positive and the cactus experience as being highly negative. Although the cactus procedure was designed to be highly unpleasant, it was not hurtful: no participant reported any injury.

This study was reviewed and approved by the Academic Committee of Chongqing Medical University. Prior to obtaining the written informed consents, detailed explanations about the study including its experimental manipulations and associated risks were provided to the participants. Also, participants were clearly informed of their freedom to withdraw from the experiment at any time, without any penalty. Each participant gave his/her written informed consent prior to the experiment and received a monetary compensation after the completion of the experiment. The current study meets the ethical standards enacted in the Declaration of Helsinki.

### Stimuli

The visual stimuli included six 200 by 150 pixels digital color images: three depicting typical sharp objects, namely, a needle, a cactus, and a bayonet; and three depicting typical soft objects, namely, a thick quilt, a pillow, and a cushion. An extra 70 students (36 males and 34 females) were recruited and asked to categorize these images as “sharp” or “soft.” No mistake was made. Before the beginning of the pairing task, the selected group of 18 participants underwent a 2 min familiarization session with the images in which they saw each image twice. Then, they had to categorize the tactile sensation associated with the objects in the image as “soft” or “sharp” tactile sensation. No error was made during this categorization task.

### Procedure

Participants were seated in a quiet room at a distance of 60 cm from the computer monitor (Hewlett-Packard liquid crystal display), and they were instructed to keep their heads still for the whole duration of the upcoming block of trials. On each block, the participants were submitted to one of the two tactile conditions: the negative or positive tactile condition. A single tactile stimulus, either soft or sharp, was presented on each block to induce a consistent positive or negative emotional state. In contrast, both types of images (i.e., of pillow and needle) were shown on every block and were assumed to have no impact on the emotional states of participants. Each block comprised 60 trials and lasted about 40 s. Each trial began with a fixation cross (“+”) displayed for 300–800 ms at the center of the screen. This duration was randomized across trials. Then, the image of a given object was presented until the participant had pressed a response key and for a maximum of 3,000 ms. Participants were instructed to indicate, as quickly and accurately as possible, whether the tactile experience associated with the object depicted in the image matched (matched condition) or did not match (mismatched condition) their current tactile experience by pressing the appropriate keyboard key. Also, the tactile stimulus was changed on each successive block to prevent tactile desensitization. Between each block, participants had to take a 300-s break during which there was no tactile stimulation.

The order of tactile stimuli was balanced: nine participants (5 male and 4 female) started with the sharp tactile (negative) block, whereas the rest started with the soft tactile (positive) block. For all selected participants, the sharp tactile sensation was associated with a highly negative emotional state; the soft tactile sensation with a highly positive emotional state; and the images with a neutral feeling. The valence of the tactile-induced emotional states (negative/positive) and the congruency of the cross-modal stimuli (matched/mismatched) were manipulated orthogonally to produce four experimental conditions: negative matched (NM; a sharp tactile stimulus and image of a sharp item), negative mismatched (NMM; a sharp tactile stimulus and image of a soft item), positive matched (PM; a soft tactile stimulus and image of a soft item), positive mismatched (PMM; a soft tactile stimulus and image of a sharp item). Participants had to complete four blocks of 60 trials each, i.e., 60 trials in each of the four experimental conditions.

### Electrophysiological Recording

Brain electrical activity was recorded from 64 scalp sites using tin electrodes mounted on an elastic cap (Brain Product, Brain Products GmbH, Stockdorfer, Gilching, Germany), with the reference on the left and right mastoids. The vertical electrooculogram (VEOG) was recorded with electrodes placed above and below the right eye, and the horizontal electrooculogram (HEOG) with electrodes placed on the outer canthi of each eye. All interelectrode impedance was maintained below 10 kΩ. The electroencephalogram (EEG) and electrooculogram (EOG) were amplified using a 0.05–100 Hz band-pass and continuously sampled at 500 Hz/channel for off-line analysis.

### Data Analysis

#### Behavioral Data Analysis

Repeated measures ANOVAs were performed on RTs and accuracy to compare the effect of the type of tactile-visual stimuli (matched vs. mismatched) and the valence of the tactile stimulation (positive vs. negative).

#### ERP Data Analysis

Brain Vision Analyzer 1.05 (Brain Products GmbH) was used to analyze ERP data. Eye movement artifacts (blinks and eye movements) were rejected off-line by using the Gratton, Coles, and Donchin algorithm (Gratton et al., 1983). This algorithm corrects for ocular artifacts by subtracting the voltages of the eye channels, multiplied by a channel-dependent correction factor, from the respective EEG channels. Trials with EOG artifacts (mean EOG voltage exceeding ± 80 μV) and those including amplifier clipping, bursts of electromyographic activity, or peak-to-peak deflection exceeding ± 80 μV were excluded from averaging. An automatic artifact rejection algorithm was used to detect trials with artifacts.

**Figure 1:**
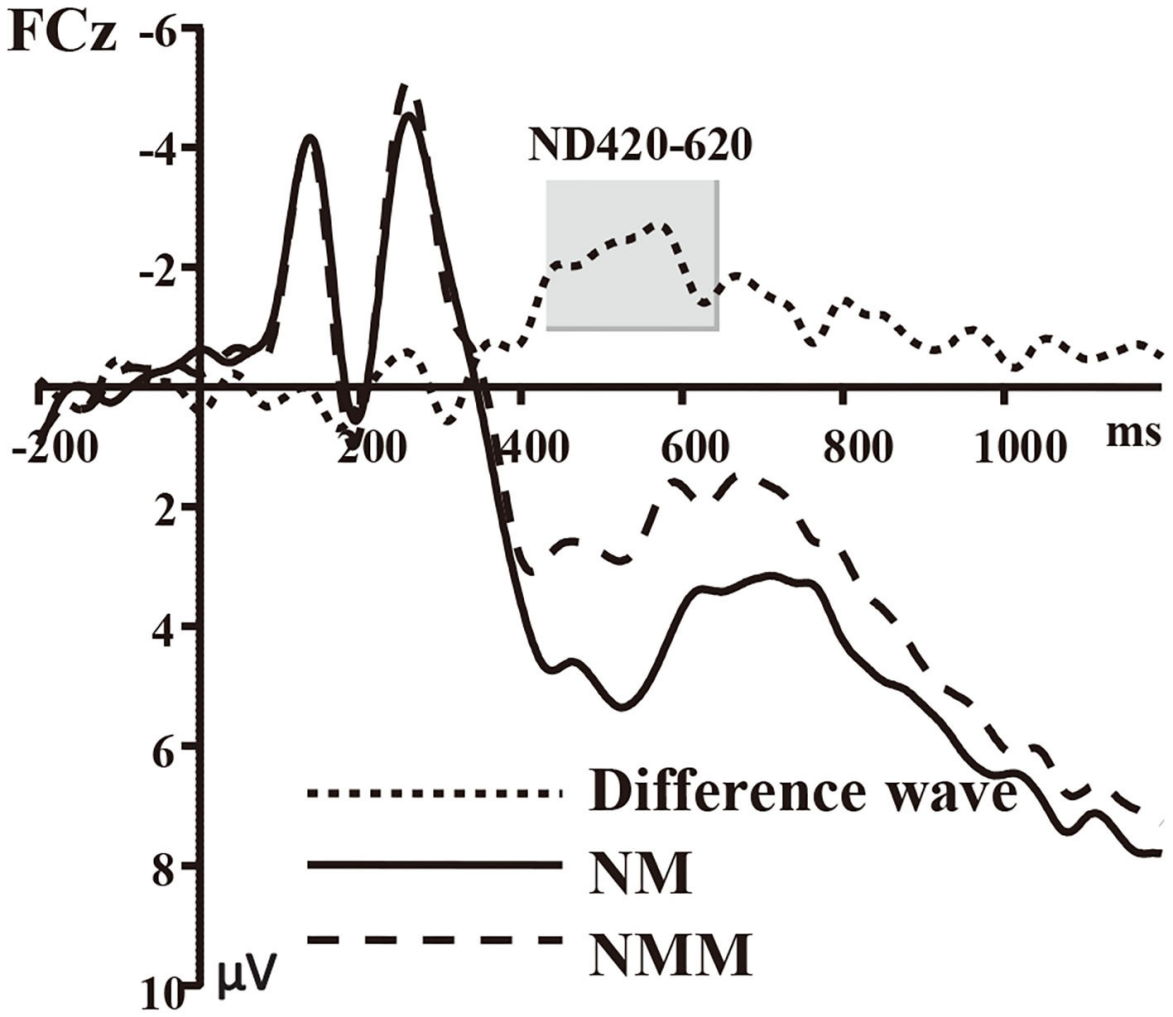
Grand average of event-related potentials recorded in the NMM and NM conditions, along with the NMM minus NM difference wave at FCz.

**Figure 2:**
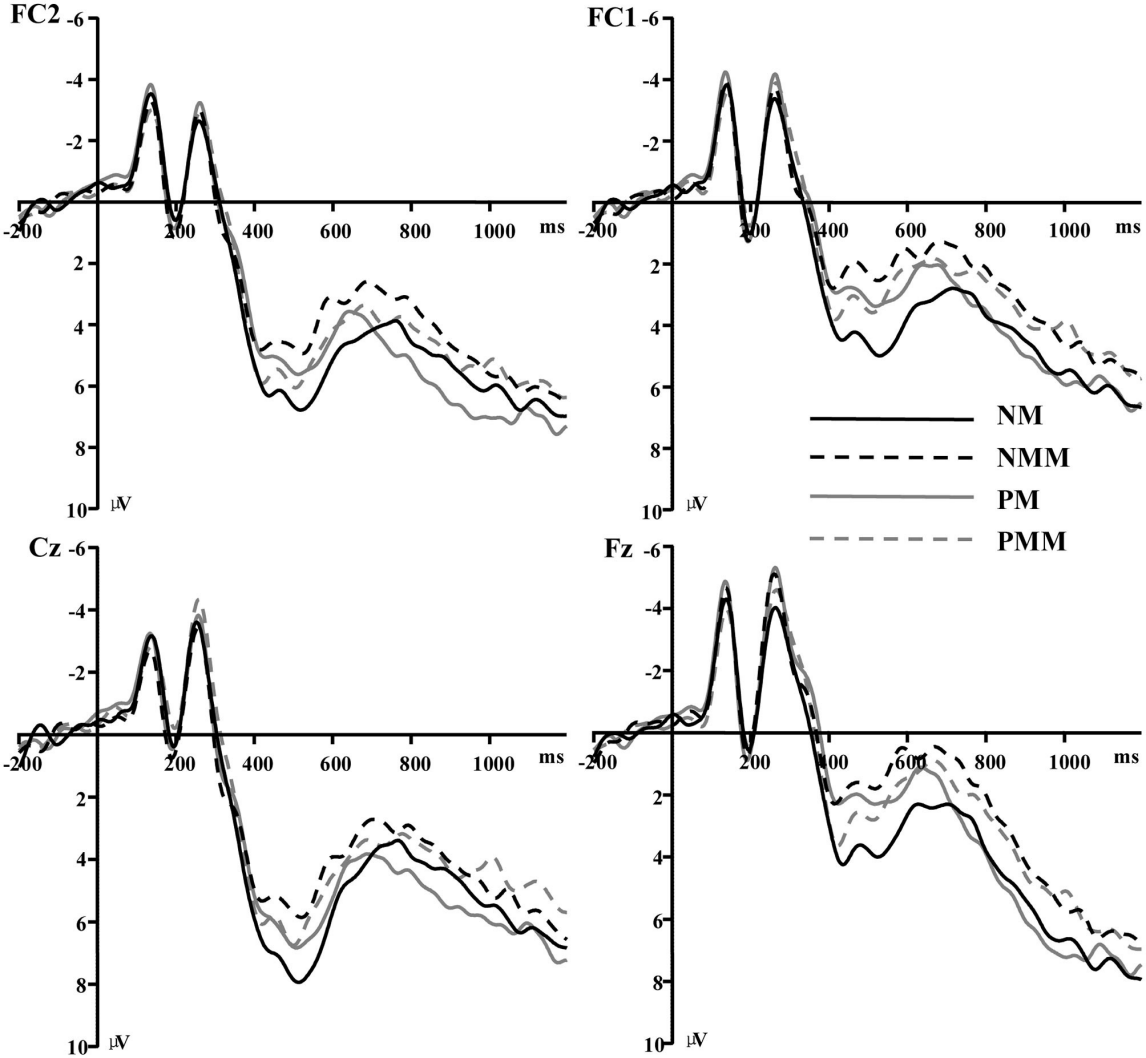
Grand average of event-related potentials recorded in the NMM, NM, PMM, and PM conditions at Fz, Cz, FC1, and FC2.

**Figure 3:**
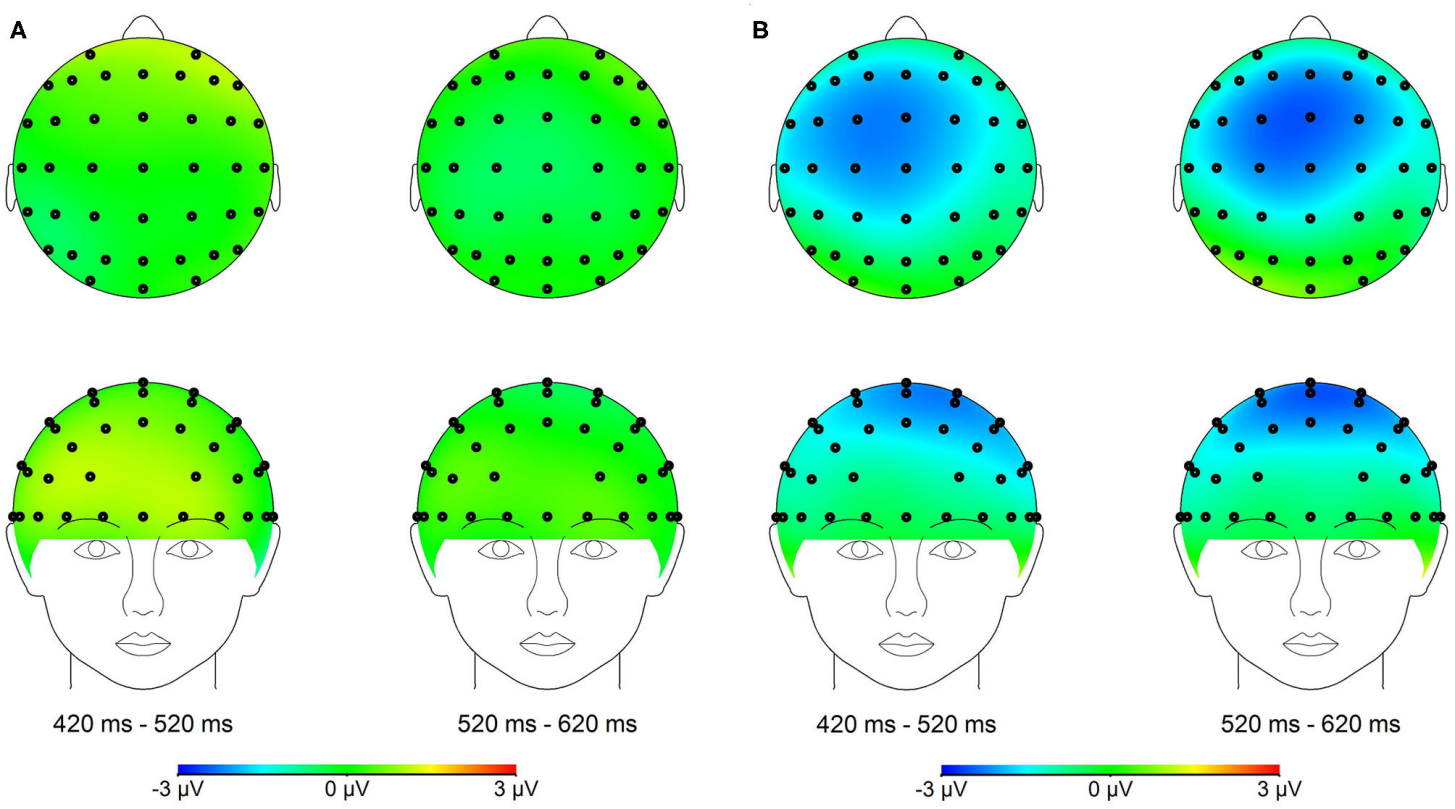
**(A)** Topographical maps of the voltage amplitudes for the PMM minus PM difference wave in the 420–620 ms time range. **(B)** Topographical maps of the voltage amplitudes for NMM minus NM difference wave in the 420–620 ms time range.

**Figure 4:**
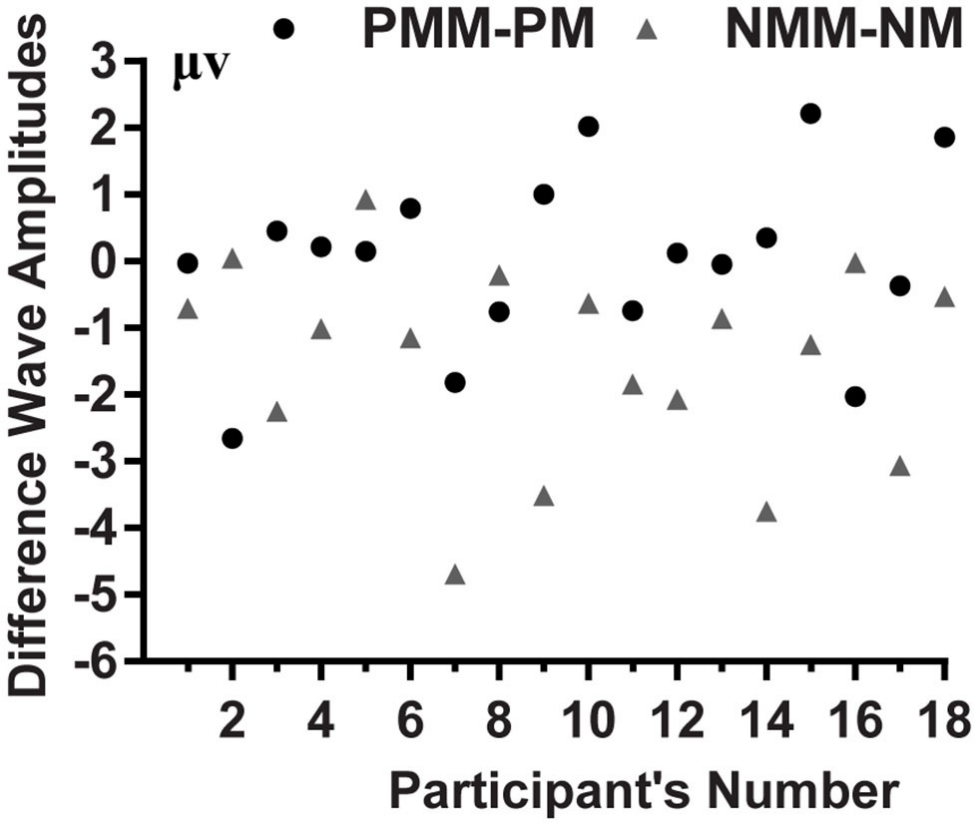
Mean voltage amplitudes of each participant's PMM minus PM, and NMM minus NM difference wave in the 420–620 ms time range.

Based on the participants' mean reaction time and previous studies (Yin et al., 2008; Wang et al., 2011; Xiao et al., 2011a), ERP segments with correct responses were averaged over an epoch of 1,400 ms going from 200 ms before visual stimulus onset to 1,200 ms after visual stimulus onset. At least 45 trials were available in each condition for a participant. Mean amplitudes computed over the 420–620 ms time window were analyzed. This time window was selected based on findings from previous studies (Yin et al., 2008; Xiao et al., 2011a) and the ERPs grand-averaged waveforms observed in the current experiment (see Figures 1–4 and Table 1).

**Table 1:** Descriptive statistics for ERP data.

**Condition**	**Mean amplitudes and SD of the ERPs between 420 and 620 ms (μV)**
Negative mismatched	2.69 ± 2.17
Negative matched	4.17 ± 2.18
Positive mismatched	3.25 ± 2.05
Positive matched	3.21 ± 2.23

Previous studies have revealed that the negative component over the fronto-central scalp region reflects the time course of conflict information processing (Wang et al., 2011). In addition, topographic maps in the present study showed larger negative difference component over the fronto-central scalp region; thus, the following 11 electrodes were chosen for statistical analysis (Fz, F1, F2, FCz, FC1, FC2, FC3, FC4, Cz, C1, and C2).

Averaged activity from the 11 electrodes computed over the 420–620 ms time window were analyzed using repeated-measures ANOVAs. The factors included in the analyses were two tactile-visual stimuli conditions (mismatched and matched) and two tactile-induced emotional state conditions (positive and negative). For all analyses, a Bonferroni correction was applied on *post-hoc* tests to correct for the family-wise error rate.

## Results

### Behavioral Results

Table 2 summarizes the average RTs and accuracy for all conditions. The effect of the type of tactile-visual stimuli (matched vs. mismatched) and the valence of the tactile-induced emotional states (positive vs. negative) on RTs and accuracy was estimated using repeated measures ANOVAs. No significant effect was found in the ANOVA on accuracy, whereas a significant main effect of tactile-visual stimuli type was found in the ANOVA on RTs [*F*_(1, 17)_ = 14.05, *p* = 0.002, η^2^ = 0.453], suggesting that it took participants more time to response in the mismatched condition than in the matched condition.

**Table 2:** Descriptive statistics for behavioral data.

**Condition**	**RT (ms)**	**Accuracy (%)**
Negative mismatched	689 ± 120	99.1 ± 1.3%
Negative matched	651 ± 106	99.4 ± 0.8%
Positive mismatched	707 ± 122	98.2 ± 2.2%
Positive matched	662 ± 122	99.0 ± 1.2%

### Electrophysiological Scalp Data

The average of mean amplitudes from the selected 11 electrodes computed over the 420–620 ms time window were analyzed using repeated-measures ANOVAs. There was a significant main effect of the tactile-visual stimuli condition within the 420–620 ms time window [*F*_(1, 17)_ = 9.21, *p* = 0.007, η^2^ = 0.351], which corresponds to a negative difference component (ND420-620). More specifically, the mismatched condition elicited a significantly smaller positive ERP deflection than did the matched condition. There was no significant main effect of the valence of the tactile-induced emotional state condition [*F*_(1, 17)_ = 1.07, *p* = 0.317]. A significant interaction was found between the tactile-visual stimuli condition and the tactile-induced emotional state condition within the 420–620 ms time window [*F*_(1, 17)_ = 10.34, *p* = 0.005, η^2^ = 0.378]. The analysis of the simple effects showed that the mismatched condition elicited significantly smaller positive ERP deflection than did the matched condition in the negative tactile-induced emotional state condition [*F*_(1, 17)_ = 17.51 *p* = 0.001, η^2^ = 0.507], whereas no difference was observed in the positive tactile-induced emotional state condition [*F*_(1, 17)_ = 0.016, *p* = 0.902]. Mean amplitudes were relatively more negative for NMM than for NM condition.

## Discussion

The present study investigated behavioral responses and ERPs associated with cross-modal conflict processing during a tactile-visual pairing task. The modulatory effect of the valence of the tactile-induced emotional states was also examined. The brain electrical activity of 18 participants was recorded with high-density EEG while they indicated whether the tactile sensation (either a constant pressure exerted on a cactus leave placed on the palm of the participant's hand, or a soft pillow placed between the participant's back and the back of his/her chair) matched or did not match the expected tactile sensation of the object (either sharp or soft) depicted in an image. The tactile-visual stimuli conditions (matched or mismatched) and the valence of tactile-induced emotional states (positive or negative) were manipulated following a 2 × 2 within-subject factorial design.

Behavioral results revealed longer reaction times when the tactile and visual stimuli did not match than when they did. Relatedly, the ERP data showed a significant mismatched vs. matched negative difference (ND420-620) waveform associated with the tactile-visual cross-modal conflict processing. A significant interaction further revealed that this ND420-620 waveform only arose during the negative—and not during the positive—tactile-induced emotional state condition. The implications of these findings are discussed next.

The mismatched condition elicited a more negative ERP component (ND420-620) than the matched condition in the negative tactile-induced emotional states condition. Similar late ND waveforms produced by incongruent cross-modal stimuli were reported in studies in which various pairs of sensory modalities were tested. For instance, Yin et al. (2008) interpreted the more negative ERP component (ND490) as a token of the audiovisual cross-modal conflict processing. Similarly, Wang et al. (2011) discovered an audiovisual mismatched minus matched negative component (ND550) related to the conflict processing of cross-modal information in children. Xiao et al. (2011a) found a mismatched minus matched ND component (ND400-600) and considered this component as a sign of incongruent taste-visual cross-modal conflict processing. Mismatched minus matched positive difference components have also been observed in rare occasions. For instance, in a study by Puce et al. (2007), participants had to indicate if audio and visual stimuli matched (e.g., human face vs. human sound) or did not match (e.g., house image vs. monkey sound). A significant positive difference component (PD400) was found between the human-face-vs.-house-sounds condition and the human-face-vs.-human-sound condition. Although the researchers interpreted this component as an index of incongruent cross-modal processing, it is hard to discount other competing hypotheses (e.g., familiarity or saliency differences) given that no positive difference component (PD400) was found in their other mismatched condition (i.e., in the human-face-vs.-monkey-sounds condition). Altogether, ERP studies using pairing tasks are accordance with our claim that the ND420-620 waveform found in our tactile-visual pairing task is related to cross-modal conflict processing.

It is also valuable to compare the ND waveforms recorded in this study with the ones observed by Xiao et al. (2011a) in a taste-visual pairing task because both used similar experimental materials and procedures. The ND waveforms (ND420-620) discovered here occurred slightly later than the ones discovered in the taste-visual cross-modal pairing task (Xiao et al., 2011a). The transmission speed of taste information far exceeds that of tactile information due to its specific processing mechanism. Taste information is conducted by nerves and almost reaches the largest speed of neural conduction, while tactile information is conducted by a series of secondary chemical reactions (Ding, 1996). Thus, one possible explanation for this later peak found in the current research could be that the speed of tactile information processing is slower than that of taste.

Another aim of the current experiment was to study the effect of positive/negative tactile-induced emotional states on tactile-visual cross-modal conflict processing abilities. The results revealed a significant ND420-620 component in the negative tactile-induced emotional state condition but not in the positive, which suggests that the valence of emotional state can modulate the ability to process tactile and visual information. This ERP difference between the positive and negative conditions might result from a facilitatory effect of positive emotional state on cognitive control abilities. Various studies have shown that cognitive control is substantially influenced by emotional states. Many studies support the idea that a positive emotional state improves cognitive flexibility and increases executive functions. For instance, Ashby et al. (1999) showed that a positive mood could improve cognitive flexibility through its effect on the dopaminergic system. In addition, our results are consistent with recent findings. Xiao et al. (2018) used the fMRI technique to study brain activation associated with cross-modal conflict processing in appetitive and aversive gustatory contexts during a taste-visual cross-modal pairing task. The results revealed that the positive emotional states induced by the delicious gustatory stimulation improves cross-modal conflict processing abilities. More recently, Xu et al. (2020) used the ERPs technique to explore the impact of the olfactory-visual cross-modal Stroop effect on brain potentials and it could be modulated by olfactory-induced affective states. The authors found an incongruent minus congruent negative difference component (ND350-550) in the negative—but not in the positive—olfactory-induced emotional state condition. This suggests that positive mood has a facilitatory effect on selective attention and cognitive control, which could decrease brain potentials related to the cross-modal conflict processing. We believe that this hypothesis could also applies to the current study, given that the pairing task and the Stroop task are very similar—and even considered as the same paradigm by some researchers (see Wang et al., 2011). Similarly, previous studies on conflict processing have also shown that the amplitude of the negative difference (ND) wave reflects the level of cognitive resources used in an interference task, that is, the amplitude of ND wave increases as the cognitive resources allocated to the task increase (Kutas and Hillyard, 1980; Xiao et al., 2011b; Yuan et al., 2011). In addition, the amplitude of ND wave is positively correlated with the difficulty of the task (Kutas and Hillyard, 1980). The fact that we observed a significant ND420-620 in the negative but not in the positive tactile-induced emotional state condition suggests that the conflict between cross-modal information was easier to resolve and required fewer cognitive resources in the positive than in the negative tactile-induced emotional states. Altogether, this evidence supports the following hypothesis: a positive sensory-induced emotional state has a facilitatory effect on cross-modal conflict processing abilities and this effect translates into a reduction of the electrophysiological activity related to the cross-modal conflict processing. The following limitations should however be noted. First, the facilitatory effect did not translate into significantly shorter RTs. It is possible that the behavioral measurements were less sensitive to the process of cross-modal conflict processing than the neurological measurements. Such lack of effect at the behavioral level was also observed in previous experiments (Luo et al., 2011; Xiao et al., 2018). Second, the positive and negative tactile conditions also differed in terms of their stimulation loci: the former being on the back, and the latter on the hand. Although there is no way to rule-out the effect of this low-level factor, the significant late ND component that was observed in this study is consistent with the results from other studies that probed different pairs of sensory modalities and used a single locus of stimulation (see Xiao et al., 2018; Xu et al., 2020).

The current study investigated the behavioral reactions and ERPs of conflict processing during a tactile-visual cross-modal pairing task. The results indicated that the mismatched condition elicited a more negative component (ND420-620) than the matched condition, which might be related to conflict processing during the incongruent tactile-visual cross-modal information processing. Interestingly, this component was found only in the negative tactile-induced emotional state condition, which suggests that the positive tactile-induced emotional state condition can improve the conflict processing abilities of tactile-visual cross-modal information. The EEG has a high temporal resolution which makes it ideal to study the time course of the tactile-visual conflict processing. However, its low spatial resolution does not allow a precise characterization of the spatial cortical activation patterns. Further experiments using other neuroimaging techniques with higher spatial resolution such as the fMRI would thus be required to investigate the brain areas associated with the tactile-visual conflict processing.

## Data Availability Statement

The datasets generated for this study are available on request to the corresponding author.

## Ethics Statement

The studies involving human participants were reviewed and approved by the Ethics Committee of Chongqing Medical University. The patients/participants provided their written informed consent to participate in this study.

## Author Contributions

XX designed this study. XX, CG, and MX performed the study. XX and CG analyzed the data and drafted the manuscript. XX, ND-R, and JJ reviewed and revised the manuscript. All authors contributed to the article and approved the submitted version.

## Conflict of Interest

The authors declare that the research was conducted in the absence of any commercial or financial relationships that could be construed as a potential conflict of interest.
